# Direct targeting of risk factors significantly increases the detection of liver cirrhosis in primary care: a cross-sectional diagnostic study utilising transient elastography

**DOI:** 10.1136/bmjopen-2014-007516

**Published:** 2015-05-02

**Authors:** David J Harman, Stephen D Ryder, Martin W James, Matthew Jelpke, Dominic S Ottey, Emilie A Wilkes, Timothy R Card, Guruprasad P Aithal, Indra Neil Guha

**Affiliations:** 1 National Institute for Health Research Nottingham Digestive Diseases Biomedical Research Unit (NDDBRU), Nottingham University Hospitals NHS Trust and University of Nottingham, Nottingham, UK; 2 NHS Rushcliffe Clinical Commissioning Group, Nottingham, UK; 3 Division of Epidemiology and Public Health, University of Nottingham, Nottingham, UK

**Keywords:** GENERAL MEDICINE (see Internal Medicine)

## Abstract

**Objectives:**

To assess the feasibility of a novel diagnostic algorithm targeting patients with risk factors for chronic liver disease in a community setting.

**Design:**

Prospective cross-sectional study.

**Setting:**

Two primary care practices (adult patient population 10 479) in Nottingham, UK.

**Participants:**

Adult patients (aged 18 years or over) fulfilling one or more selected risk factors for developing chronic liver disease: (1) hazardous alcohol use, (2) type 2 diabetes or (3) persistently elevated alanine aminotransferase (ALT) liver function enzyme with negative serology.

**Interventions:**

A serial biomarker algorithm, using a simple blood-based marker (aspartate aminotransferase:ALT ratio for hazardous alcohol users, BARD score for other risk groups) and subsequently liver stiffness measurement using transient elastography (TE).

**Main outcome measures:**

Diagnosis of clinically significant liver disease (defined as liver stiffness ≥8 kPa); definitive diagnosis of liver cirrhosis.

**Results:**

We identified 920 patients with the defined risk factors of whom 504 patients agreed to undergo investigation. A normal blood biomarker was found in 62 patients (12.3%) who required no further investigation. Subsequently, 378 patients agreed to undergo TE, of whom 98 (26.8% of valid scans) had elevated liver stiffness. Importantly, 71/98 (72.4%) patients with elevated liver stiffness had normal liver enzymes and would be missed by traditional investigation algorithms. We identified 11 new patients with definite cirrhosis, representing a 140% increase in the number of diagnosed cases in this population.

**Conclusions:**

A non-invasive liver investigation algorithm based in a community setting is feasible to implement. Targeting risk factors using a non-invasive biomarker approach identified a substantial number of patients with previously undetected cirrhosis.

**Trial registration number:**

The diagnostic algorithm utilised for this study can be found on clinicaltrials.gov (NCT02037867), and is part of a continuing longitudinal cohort study.

**Strengths and limitations of this study**

- The current study provides an alternative diagnostic algorithm to the usual flawed concept of investigating patients for liver disease on the basis of elevated liver function enzymes. We introduced a novel algorithm that targets established risk factors for chronic liver disease in a primary care setting. Patients were investigated utilising simple and cheap blood-based biomarkers followed by transient elastography (a validated biomarker of liver fibrosis).
- Patients with common lifestyle-related risk factors for chronic liver disease (including hazardous alcohol use and type 2 diabetes) were identified using a widely utilised prospective primary care database in the UK, from two general practices with an adult population of 10 479 patients. Our patient selection method, and subsequent investigation algorithm, would therefore be easy to adopt at other primary care sites in the UK.
- Further study is required in a number of areas to address limitations of the current study, including validation of the diagnostic algorithm in other regions of the UK, investigation using alternative simple fibrosis markers or transient elastography and health-economic analysis of earlier identification of chronic liver disease.

## Introduction

Current strategies to identify liver disease in the general population rely on the use of liver function enzyme blood tests, which are non-specific markers of liver injury. In the UK, persistently elevated liver enzymes typically trigger referral to hospital-based gastroenterology and hepatology clinics, where liver biopsy is the reference standard investigation for staging of hepatic fibrosis. This is an inadequate strategy, as the absence of symptoms in early stages of liver disease, and poor sensitivity of liver function blood tests to detect hepatic fibrosis, often result in late diagnosis. A recent study using data from a large community database, the clinical practice research datalink, suggested that 50% of patients with cirrhosis are given their initial diagnosis of liver disease after the first hospitalisation with decompensation, which substantially impaired prognosis independent of stage of cirrhosis, versus patients who are diagnosed while ambulatory (HR=2.78, 95% CI 2.53 to 3.06).^1^ By contrast, many patients referred for investigation of abnormal liver tests have no evidence of significant liver disease when investigated in hospital. A large study from Tayside, Scotland,^2^ has shown that at least 25% of a community population will have their liver function tests (LFTs) checked in a decade and around a third will have at least one abnormal value. While abnormal transaminases were predictive of liver disease (HR=4.2), the actual rate of detection was very low with only 3.9% of those with abnormal transaminases being diagnosed with liver disease within 5 years of the abnormal test. Subsequently, the BALLETS study^3^ has shown convincing evidence that most abnormal LFTs in primary care are due to non-alcoholic fatty liver disease (NAFLD) or alcohol, and the yield for detecting other parenchymal liver disease was only 3%.

These inefficient diagnostic algorithms have contributed to the fact that liver-related deaths have continued to rise in the UK,^4^ in stark contrast to other major causes of death.^5^ Liver cirrhosis is now the third commonest cause of premature death (persons aged less than 55 years).^5^ Given the majority of chronic liver disease results from lifestyle-related risk factors that are amenable to intervention, many deaths from chronic liver disease are preventable. In order to tackle the rising cirrhosis prevalence and mortality, we need an investigation strategy that results in early detection of hepatic fibrosis while patients are still asymptomatic, and which is both economically efficient and acceptable to patients. Targeting risk factors for chronic liver disease rather than abnormal blood tests alone, performed directly in a community setting, is one such approach that is fundamentally different to current diagnostic pathways. In a community setting, liver biopsy is not a practical reference standard for liver disease stratification as it is invasive and requires specialist provision.^6^ Numerous non-invasive alternatives to liver biopsy for fibrosis staging have been validated in hospital populations, but require further evaluation in primary care before widespread adoption into investigation algorithms for the general population.

Despite the widespread recognition that significant liver disease exists in the context of normal LFTs, current management algorithms are still dependent on abnormal liver enzymes to initiate investigation. Our aim was to challenge this flawed concept by testing the feasibility of a novel diagnostic algorithm that directly targeted risk groups (including alcohol and diabetes), rather than relying on LFTs. Importantly, we designed a pathway that integrated non-invasive diagnostic tests and liver specialists within a community setting. The pathway used simple tests followed by a validated test of liver fibrosis (transient elastography, TE). We hypothesised this approach would detect a substantial number of hitherto undiagnosed cases of chronic liver disease.

## Materials and methods

### Study setting

This was a prospective study with recruitment from two suburban general medical practices in Rushcliffe, Nottingham, UK (the least deprived borough in Nottingham), with a total patient population of 12 368. The study ran for 14 months from February 2012 to April 2013; local regulatory approval was obtained (10/HO405/8 and 13/EM/0123), and written informed consent was gained from patients. Both practices utilise the SystmOne general practice records system (TPP, UK) for live recording of clinical, anthropometric and biochemical patient data. Data are stored as searchable numeric data or prospectively coded with ‘Read codes’ (clinical encoding of numerous parameters including patient demographics, diagnoses, clinical signs and laboratory test results).

### Study patient selection

From the electronic general practice records, we identified adult patients (aged 18 years or older) with selected risk factors for lifestyle-related chronic liver disease at the start of the study period. Patients were eligible for study inclusion regardless of previous liver function blood test results, if identified with one or more of the following chronic liver disease risk factors:

- Hazardous alcohol use—defined as >14 units per week ethanol consumption for women and >21 units per week ethanol consumption for men, or alcohol AUDIT questionnaire score ≥8, or presence of Read codes related to alcohol misuse.
- Type 2 diabetes—presence of Read codes related to type 2 diabetes (a full code list is available from the authors on request).
- Raised alanine aminotransferase (ALT)—as a comparator group, patients with persistently elevated serum ALT levels (defined as ≥2 consecutive occasions above local laboratory cut-offs) without type 2 diabetes or hazardous alcohol use were assessed. In order to ensure only patients with alcohol-related liver disease or NAFLD were investigated, other causes of chronic liver disease were excluded by specific testing of chronic viral hepatitis B and C, autoimmune liver diseases, haemochromatosis and common genetic liver diseases, and no other relevant reason for transaminase elevation (eg, congestive cardiac failure, medication use). Local laboratory cut-offs denoting elevated ALT are >35 U/L for women and >45 U/L for men.

Identified patients were excluded from the study a priori if: (1) there was definitive evidence of hepatic fibrosis or cirrhosis from previous investigation, (2) there was a contraindication to TE (pregnancy, indwelling cardiac device) or (3) patients were unable to consent to investigation, or were housebound and could not attend the community practice.

### Diagnostic algorithm

**Figure 1:**
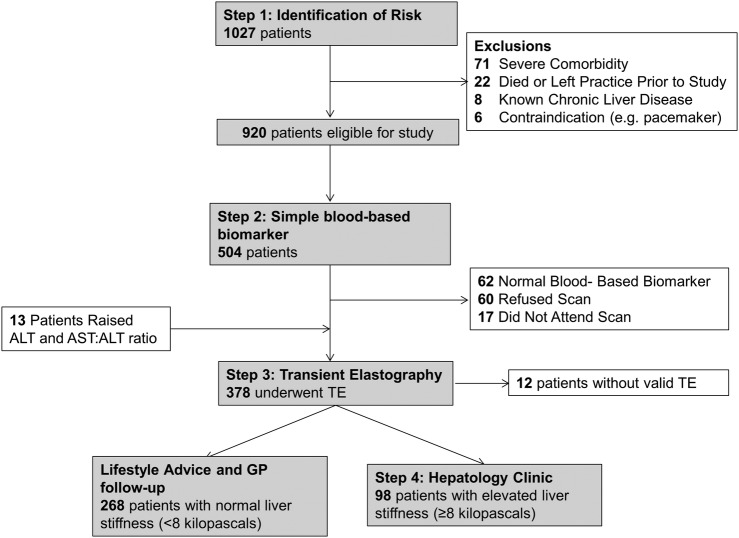
Diagnostic algorithm and patient flow chart through non-invasive biomarker pathway. ALT, alanine aminotransferase, AST, aspartate aminotransferase; GP, general practitioner; TE, transient elastography.

Patients identified with the defined risk factors were invited to the diagnostic algorithm (see figure 1). Patients with type 2 diabetes were invited opportunistically at their diabetes annual review. Patients with hazardous alcohol use were invited opportunistically during primary care appointments or via letter where they did not undergo a consultation during the study period. Patients in the raised ALT subgroup were prospectively referred by the investigating general practitioner (GP).

Patients initially underwent a simple blood-based biomarker, with a high negative predictive value to accurately rule out hepatic fibrosis and therefore avoid the need for further tests. Hazardous alcohol users had the aspartate aminotransferase (AST):ALT ratio measured, a simple and cheap blood biomarker that has been shown to reflect disease severity in a number of chronic liver diseases, including alcoholic liver disease,^7^ autoimmune liver disease^8^ and hepatitis C.^9^ As there are limited published data on diagnostic thresholds of AST:ALT ratio identifying alcohol-related liver fibrosis, a conservative AST:ALT cut-off of 0.8 was used to rule out hepatic fibrosis. Patients with type 2 diabetes or persistently raised ALT had the BARD score,^10^ which is a simple weighted score of body mass index (BMI) ≥28 kg/m^2^, AST:ALT ratio ≥0.8 and type 2 diabetes, measured. A BARD score of less than 2 in hospital populations of NAFLD has a high negative predictive value to rule out significant hepatic fibrosis.^10–13^ We therefore considered that a score of ≥2 indicated an increased risk. Patients with a normal simple biomarker test result did not proceed down the algorithm.

Patients with a high simple biomarker result underwent TE at the community practice. TE is a non-invasive imaging method that calculates liver stiffness via measurement of the propagation of an elastic shear wave through the liver substance. TE accurately predicts liver biopsy findings of fibrosis and cirrhosis in all major aetiologies of chronic liver disease.^14^ TE was performed by one of three trained nurses, all of whom had performed more than 50 examinations in the hospital prior to the start of study, using the portable Fibroscan FS402 device (Echosens, Paris). The technique for liver stiffness measurement using elastography has been previously described in detail.^15^ The median value of successful measurements, measured in kilopascals (kPa) was representative of liver stiffness. A scan failure was defined as the inability to obtain 10 valid elastography measurements on a single patient. A successful TE result was deemed unreliable if liver stiffness was ≥7.1 kPa and the IQR/median was greater than 0.3 as per recent guidance.^16^ ^17^ Patients with a BMI of greater than 35 kg/m^2^ underwent TE at the hospital using the XL probe (FS502 device), due to high rate of scan failures using the standard M probe above this BMI threshold.^18^ All patients, regardless of TE threshold result, received lifestyle advice from the nurses and were given the British Liver Trust ‘Looking After Your Liver’ leaflet.

A TE threshold of 8.0 kPa or greater, which has been previously shown to accurately determine presence of hepatic fibrosis during community screening,^19^ was used to define elevated liver stiffness, and hence clinically significant liver disease. Patients with high liver stiffness results, including high but unreliable acquisitions, were reviewed by a visiting consultant hepatologist in the community. Where appropriate, further investigations including ultrasonography, liver biopsy and enrolment into cirrhosis surveillance programmes, were organised after this consultation. Cirrhosis was definitively diagnosed in all cases based on established clinical, radiological (including TE result) and/or histological assessment. Local cut-offs for elevated ALT (>35 U/L for women and >45 U/L for men), suggested alternative cut-offs from the literature^20^ (>19 U/L for women and >30 U/L for men), and simple serum fibrosis scores AST:platelet ratio index (APRI) (>1.5)^21^ and FIB4 (>3.25)^22^ were compared for identifying elevated liver stiffness and cirrhosis in the study population.

### Statistics

Statistical analysis was performed using SPSS V.19.0 (IBM). Categorical data are presented as numbers (percentage). Continuous data are presented as mean (SD) for parametric data and medians (range) for non-parametric data. Anthropometric and biochemical data were compared between patients with normal and elevated liver stiffness—continuous variables were compared using the two sample t test for parametric variables and Mann-Whitney test for non-parametric variables. Categorical variables were compared using χ^2^ test, or Fisher's exact test where appropriate.

## Results

### Study population

The total patient population of the two participating general medical practices was 12 368, of which 10 479 patients were adults. In total, 5922 adult patients (56.5%) had alcohol consumption documented, with 6.3% of the total GP population (658 patients) meeting our definition for hazardous alcohol use. The adult prevalence of type 2 diabetes was 3.7% (390 patients). Both of these risk factors were found in 21 patients and thus 1027 patients were identified for the study. We excluded 107 patients and therefore 920 were invited to the study (see figure 1). Of the excluded patients eight had prior definitive staging of liver disease due to alcohol (3 patients), hepatitis B (2), non-alcoholic steatohepatitis (NASH; 1), haemochromatosis (1) and primary biliary cirrhosis (1). Overall, 504 patients (54.8%) underwent the simple blood-based biomarker; the baseline characteristics for these patients compared with all adult patients in the primary care practices are shown in table 1. Patients who underwent the simple blood-based biomarker were older than the comparative total adult primary care population, and were more likely to be male, obese and have ischaemic heart disease. Compared with the 416 patients who did not undergo liver disease screening, the 504 attenders were significantly older (mean age 60.4 vs 40.3 years; p<0.001), but gender was no different (male gender 69.8% vs 69.8%; p=1.00).

**Table 1:** Baseline characteristics of all adult patients at studied general practice sites compared with patients undergoing study

Variable	All adult GP patients (n=10 479)	Patients undergoing study* (n=504)	p Value
Age, n (%) (years)			
18–30	2100 (20.4)	20 (4.0)	**<0**.**001**
31–40	2469 (23.6)	45 (8.9)	**<0**.**001**
41–50	1903 (18.2)	66 (13.1)	**0**.**005**
51–60	1331 (12.7)	94 (18.7)	**<0**.**001**
61–70	1287 (12.3)	131 (26.0)	**<0**.**001**
71–80	781 (7.5)	103 (20.4)	**<0**.**001**
>80	608 (5.8)	45 (8.9)	**0**.**005**
Male gender, n (%)	5131 (49.0)	352 (69.8)	**<0**.**001**
Body mass index, n (%)			
25–29.9	2835 (27.1)	201 (39.9)	**<0**.**001**
≥30	1320 (12.6)	160 (31.7)	**<0**.**001**
Missing	1363 (13.0)	17 (3.4)	**<0**.**001**
Hazardous alcohol use, n (%)	658 (6.3)	247 (49.0)	**<0**.**001**
Type 2 diabetes, n (%)	390 (3.7)	276 (54.8)	**<0**.**001**
Ischaemic heart disease, n (%)	423 (4.0)	65 (12.9)	**<0**.**001**
Hypertension, n (%)	1521 (14.5)	215 (42.7)	**<0**.**001**
Hyperlipidaemia, n (%)	2117 (20.2)	310 (61.5)	**<0**.**001**

*Patients undergoing study refers to all patients having the simple blood biomarker step of stratification. Categorical variables are displayed as n (%) and compared using χ^2^ test.

p Values reaching statistical significance (p≤0.05) are shown in bold.

GP, general practitioner.

Blood test uptake differed significantly between patients with type 2 diabetes and hazardous alcohol users (91.4% vs 37.6%, p<0.001). Normal blood-based biomarker results were seen in 62 patients (12.3%). In total, 378 patients underwent TE, of whom portable M-probe readings were performed in 361 patients (95.5%), while XL probe readings were performed in the remaining 17 patients. It was not possible to obtain a valid liver stiffness measurement in 12 patients (3.2%), and a further 24 patients (6.3%) had an unreliable measurement.

### Chronic liver disease risk factors in those with raised blood-based biomarkers

**Figure 2:**
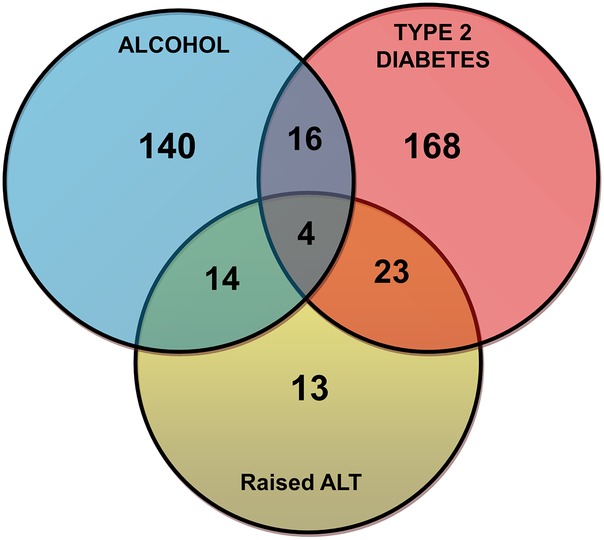
Distribution of chronic liver disease risk factors for all patients (n=378) undergoing transient elastography examination (ALT, alanine aminotransferase).

Hazardous alcohol use was present in 174 patients (46.0%), type 2 diabetes in 211 patients (55.8%) and raised ALT in 54 patients (14.3%; includes 13 patients without hazardous alcohol use or type 2 diabetes). Overall, 57 patients (15.1%) had more than one chronic liver disease risk factor (see figure 2). Baseline characteristics for all patients undergoing TE and the individual risk factor groups are shown in table 2.

**Table 2:** Baseline population characteristics of 378 patients with raised blood biomarker result undergoing TE

Variable	All patients undergoing TE (n=378)	Hazardous alcohol use* (n=174)	Type 2 diabetes* (n=211)	Raised ALT* (any case) (n=54)
Age, years	61.8 (15.0)	54.8 (14.9)	68.5 (11.3)	57.0 (11.5)
Male, n (%)	255 (67.5)	138 (79.3)	130 (61.6)	32 (59.3)
Body mass index, kg/m^2^	28.53 (5.25)	26.5 (4.2)	30.0 (5.2)	31.1 (5.7)
Obesity, n (%)	130 (34.4)	32 (18.4)	100 (47.4)	29 (53.7)
Hypertension, n (%)	173 (45.8)	47 (27.0)	134 (63.5)	23 (42.6)
Metabolic syndrome, n (%)	117 (31.0)	18 (10.3)	105 (49.8)	24 (44.4)
Ischaemic heart disease, n (%)	53 (14.0)	11 (6.3)	44 (20.9)	7 (13.0)
Type 2 diabetes, n (%)	211 (55.8)	20 (5.7)	211 (100)	27 (50.0)
Hazardous alcohol use, n (%)	174 (46.0)	174 (100)	20 (9.5)	18 (33.3)
Platelets, 10^9^/L	240.66 (62.45)	240.2 (54.8)	239.6 (65.0)	240.2 (64.6)
Creatinine, µmol/L	90.21 (23.65)	87.7 (20.8)	92.9 (27.9)	85.2 (15.0)
Bilirubin, µmol/L	11.97 (5.04)	12.4 (4.4)	11.7 (5.3)	11.2 (6.2)
Albumin, g/L	37.78 (2.92)	38.2 (2.9)	37.5 (3.0)	38.0 (2.8)
ALT, IU/L	28.09 (19.06)	28.4 (21.4)	27.2 (15.7)	60.4 (30.6)
AST, IU/L	27.91 (18.97)	30.7 (24.6)	25.0 (11.0)	52.0 (39.6)
AST:ALT ratio	1.09 (0.36)	1.16 (0.35)	1.03 (0.37)	0.86 (0.24)

Numerical variables are displayed as mean (SD), categorical variables are displayed as n (%).

*Patient numbers refer to patients fulfilling the single risk factor alone (see figure 2).

ALT, alanine aminotransferase, AST, aspartate aminotransferase; TE, transient elastography.

### Significant liver disease detection

**Figure 3:**
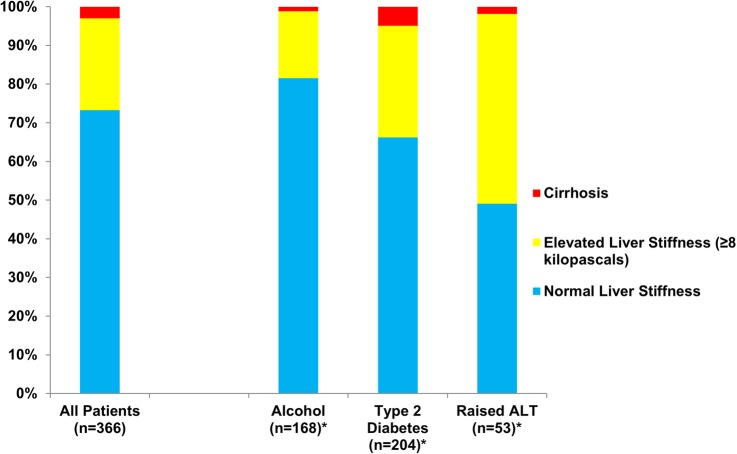
Transient elastography results of patients with successful liver stiffness acquisition (n=366) (ALT, alanine aminotransferase).

Valid liver stiffness acquisition was possible in 366 patients (96.8%). A new diagnosis of clinically significant liver disease was made in 98 patients (26.8%) with valid TE measurement. This represents a substantial increase in diagnoses for these practices. A significantly greater percentage of patients with type 2 diabetes had elevated liver stiffness compared with hazardous alcohol users (34.0% vs 18.3%, p≤0.001; figure 3). Patients with more than one defined risk factor (n=53 with successful TE measurement) had a significantly greater percentage of elevated liver stiffness compared with patients with type 2 diabetes alone (49.1% vs 29.5%, p=0.01) or with hazardous alcohol use alone (49.1% vs 14.1%, p<0.001). In the subgroup of patients with raised ALT but neither hazardous alcohol use nor type 2 diabetes (figure 2), elevated liver stiffness was observed in 4/13 (30.8%) patients with raised ALT but neither hazardous alcohol use nor type 2 diabetes. A diagnosis of NASH with early fibrosis was assigned in all four cases.

Patients with elevated liver stiffness (n=98) were older, had a higher BMI and higher prevalence of the metabolic syndrome than those with normal liver stiffness (n=268). On laboratory testing, patients with elevated liver stiffness had lower platelet count, greater prevalence of thrombocytopaenia, higher serum ALT and lower AST:ALT ratio than patients with normal liver stiffness (see online supplementary table S1). The simple serum fibrosis scores for APRI and FIB4 were significantly higher in patients with elevated liver stiffness. However, these markers were only elevated in 2.0% and 10.2% of patients with elevated liver stiffness, respectively.

Of 98 patients referred for community assessment by the hepatologists, liver biopsy was performed in 25 patients for whom there was diagnostic uncertainty on review of clinical data and TE results. Median biopsy length was 13 mm (IQR 7.5–18.0), and 12/25 patients had cores ≥15 mm. Hepatic fibrosis was confirmed in 20 patients undergoing liver biopsy; the remainder all had steatohepatitis (see online supplementary figure S1). Overall, 11 patients were newly diagnosed with liver cirrhosis during the study period based on clinical, radiological and/or histological assessment; 4 of these patients had additional evidence of portal hypertension (see online supplementary table S2). Prior to starting the study, only eight patients were known to have cirrhosis in this primary care population. The new observed cirrhosis prevalence of 19 patients after the study period therefore represents a 140% increase compared with before the study; usual care methods missed the majority of patients with very advanced liver disease.

For local ALT cut-offs (>35 U/L for women and >45 U/L for men), 72.4% of patients with elevated liver stiffness, 60% with liver fibrosis on biopsy and 90.9% with liver cirrhosis had normal ALT levels, and would have been missed by standard diagnostic algorithms. Using conservative cut-offs (>19 U/L for women and >30 U/L for men) would still have missed 41.8% of patients with elevated liver stiffness and 18.2% with cirrhosis (see table 3). Simple serum score thresholds of APRI >1.5 and FIB4 >3.25 would have detected 0 and 2 (18.2%) patients with cirrhosis, respectively.

**Table 3:** Serum alanine aminotransferase (ALT) levels of 366 patients with successful liver stiffness acquisition

Liver stratification	Raised ALT local laboratory*	Normal ALT local laboratory*	Raised ALT 19/30†	Normal ALT 19/30†
Normal liver stiffness (n=268)	26 (9.7%)	242 (90.3%)	88 (32.8%)	180 (67.2%)
Elevated liver stiffness (n=98)	27 (27.6%)	71 (72.4%)	57 (58.2%)	41 (41.8%)
Cirrhosis (n=11)	1 (9.1%)	10 (90.9%)	9 (81.8%)	2 (18.2%)
**Serum ALT levels of 25 patients with elevated liver stiffness undergoing subsequent percutaneous liver biopsy**				
No hepatic fibrosis (n=5)	1 (20%)	4 (80%)	3 (60%)	2 (40%)
Any hepatic fibrosis (n=20)	8 (40%)	12 (60%)	15 (75%)	5 (25%)

Numerical values are displayed as n (%).

*Locally utilised cut-offs: ALT >35 IU/L for women and >45 IU/L for men.

†Alternative cut-offs: ALT >19 IU/L for women and >30 IU/L for men. Values are displayed as n (%) in all cases.

## Discussion

### Statement of principal findings

This study uses a novel approach to detect clinically significant but asymptomatic chronic liver disease, based on: (A) directly targeting established risk factors and not simply using LFTs and (B) the integration of validated non-invasive tests of liver fibrosis and liver specialists within a community setting. The utilisation of a simple, low-cost algorithm detected a large number of previously undiscovered subclinical but significant chronic liver disease cases, including 98 patients with elevated liver stiffness (26.8% of all those with successful liver stiffness measurements), and the number of observed cases of liver cirrhosis in the primary care population of 10 479 adult patients from which our high-risk patients were drawn was increased by 140% compared with before the study. The study highlights the inadequacy of liver function enzymes as a stratification tool in primary care: 72% of patients with elevated liver stiffness, 60% with liver fibrosis on biopsy and 91% diagnosed with cirrhosis had normal ALT. In the absence of liver function abnormalities, or specific liver-related symptoms, their disease would have been missed by the standard diagnostic algorithms. We have demonstrated this diagnostic approach is feasible to implement and acceptable to patients, with more than 95% of investigations occurring in the community setting and non-attendance rates for TE appointments of less than 5%. This nurse-led TE service, which we originally established in the hospital setting,^23^ has now been implemented in the community with equivalent quality assurance as evidenced by successful TE acquisition in 96.8% and reliable measurements in 90.5% of cases.

### Strengths and weaknesses of the study

This study is the first to utilise blood biomarkers and TE to stratify patients at risk of liver disease in a community population of the UK. We have utilised a novel diagnostic algorithm, which is rapidly applicable in clinical practice, in a large community population of more than 10 000 adults. Targeting patients with well-defined risk factors for liver disease resulted in a high pretest probability of detecting hepatic fibrosis, with 26.8% of patients with TE results exceeding the threshold for clinically significant liver disease. Of 25 patients undergoing confirmatory liver biopsy, 80% had histological evidence of fibrosis. It is possible to argue that the remaining biopsies exposed patients to unnecessary risks of liver biopsy, although these five patients had histological evidence of steatohepatitis, suggesting a high risk of future progression to hepatic fibrosis or cirrhosis. This is a far greater diagnostic yield than traditional algorithms using elevated liver enzymes alone to stratify fibrosis risk,^2^ ^3^ and is likely therefore to be a cost-efficient approach; formal health-economic modelling studies are required to confirm this. The use of simple and inexpensive tests as the initial diagnostic, followed by more specialist tests, rationalised resource use and enhanced the feasibility of stratifying the large community population. Moreover, we extrapolated data from hospital-based studies and used conservative thresholds for the diagnostic tests. These prior studies demonstrated a high negative predictive value (≥93%)^11^ ^14^ for ruling out significant fibrosis using our chosen fibrosis markers. In a community population, where the disease prevalence is lower than prior hospital-based studies, we would expect their negative predictive value to be higher (albeit in exchange for a reduction in positive predictive value). Thus, we believe it is unlikely that we have missed significant disease within the risk groups that we have stratified. However, long-term follow-up of these patients will be necessary to confirm this. As it is clear that the type of risk factor and presence of multiple risk factors alter the diagnostic yield, a further strength of the pathway is that it can be applied to diverse risk factors, with either the tests or thresholds adjusted depending on the initial risk factor(s), and this improves the generalisability of our pathway to other healthcare regions.

However, there are limitations to discuss from our approach. We investigated patients from specific medical practices within a distinct sociodemographic area of the UK. It is possible that both patient attendance and the detection of clinically significant liver disease may differ elsewhere, and further study of our algorithm in other regions is necessary. The pragmatic study design, both in terms of biomarker selection and investigation of selected risk factors for liver disease, means we also cannot formally assess the sensitivity of the algorithm, or the total fibrosis and cirrhosis prevalence, in this community population. Alcohol use was only screened in 57% of this community population, which is lower than the approximately 70% documentation of alcohol intake in primary care practices in the East Midlands region.^24^ We also cannot account for those patients who have under-reported their alcohol intake to be within normal limits. Furthermore, additional risk factors such as obesity and the metabolic syndrome, while incorporated partially in this pathway (eg, BARD score) were not specifically included, to ensure feasible stratification of the defined at-risk patient groups during the time of the study period. Taken together, the detected prevalence of significant disease, including cirrhosis, may represent an underestimate and the presence of cirrhosis in the community is likely to be higher than we report. Also of note, patient uptake of screening was not optimal (55% of targeted patients were investigated with the algorithm). However, this is comparable to uptake rates of colorectal cancer screening,^25^ ^26^ and greater than other community liver disease stratification studies, where screening uptake is explicitly reported.^27–29^ In the UK, patients with diabetes undergo an annual health check, which made them more accessible for opportunistic invitation for screening, and this is one explanation for this significant difference in uptake compared with patients with hazardous alcohol use (91% vs 38% undergoing investigation, respectively). Lastly, there is scope to improve our diagnostic algorithm further. In total, only 12% of patients were excluded from TE by the simple blood-based biomarkers. Future studies using alternative simple serum fibrosis tests, including APRI and FIB4, compared with TE alone, are warranted to develop an optimal significant liver disease detection algorithm in the community.

### Strengths and limitations in relation to other studies

Previous studies of chronic liver disease stratification in the general population support our findings of an appreciable burden of previously undetected chronic liver disease. A study of presumed well army personnel and families in the USA^30^ screened 328 participants with ultrasound and liver biopsy for NAFLD. This approach identified a high prevalence of both NASH (12.2%) and significant fibrosis (2.7%). An alternative, non-invasive approach using TE was employed to screen a defined general population for significant liver disease in France.^19^ TE was performed in 1358 asymptomatic patients over the age of 45 from a well person clinic. An elevated reading, above a validated threshold of 8 kPa, was found in 7% of the cohort and nine cases of cirrhosis were detected. A further study of 7463 patients aged over 40 years and investigated sequentially with Fibrotest (a serum fibrosis marker) and TE, detected a fibrosis prevalence of 2.8% and cirrhosis prevalence of 0.3%.^31^ Importantly, these prior studies used an unselected approach to detect liver disease in the community. We deliberately focused our diagnostic tests on individuals with risk factors for liver disease. The pretest probability of detecting disease is increased using this approach and this is evidenced by the fact that 26% of our cohort undergoing TE had elevated liver stiffness readings compared with 7% in the Roulot *et al*^19^ study. A risk factor in combination with the elevated biomarker tests also rationalises the use of liver biopsy with a high yield of clinically significant liver disease. In our study, the positive predictive value of selected patients undergoing liver biopsy was 80% for detecting any fibrosis. This compares favourably to the 18% positive predictive value of finding any fibrosis on biopsy following investigation of raised liver enzymes in seronegative individuals.^32^

A recent study in the UK investigated 1118 patients with elevated liver enzymes in primary care; in those with suspected NAFLD, 7.6% were found to have significant disease based on an elevated NAFLD fibrosis score.^3^ An important finding from our study is that normal ALT levels, within the local laboratory range, were found in 72% of patients with elevated liver stiffness and 91% of patients with cirrhosis. Thus, the reliance on abnormal liver enzymes to detect chronic liver disease, which represents standard clinical practice, is inherently flawed. In NAFLD, the presence of significant liver disease in the context of normal enzymes is well documented. The entire spectrum of NAFLD severity has been shown to exist in patients with normal liver enzymes; multiple studies have observed no difference in disease severity between this group and those with abnormal liver enzymes.^33–35^ Interestingly, if more conservative thresholds are utilised in our population at the 19/30 U/L cut-off, as advocated by the work of Prati *et al*,^20^ then a normal ALT is still observed in 42% of individuals with elevated liver stiffness and in 18% of those with cirrhosis. In those patients with an elevated ALT, 13 patients (24%) did not have type 2 diabetes or hazardous alcohol use as a comorbidity. Only 4 out of these 13 patients had an abnormal TE reading. Thus, our work strongly supports the concept that the risk for chronic liver disease is better encapsulated by the underlying risk factor as opposed to liver enzyme tests alone.

### Implications for clinicians and policymakers

The utilisation of a simple, low-cost and patient acceptable algorithm in the community resulted in the detection of a large number of cases of previously unidentified chronic liver disease. While the detection of these new cases is currently of unproven benefit, there are likely to be a number of advantages. Early identification of asymptomatic liver disease is likely to reduce future liver-related morbidity and mortality. Interventions for lifestyle-related disease risk factors form established treatments (such as brief interventions for alcohol, or dietary and exercise advice), can be performed in a community setting^36^ ^37^ and are cost-effective.^38^ ^39^ A decision analysis study that modelled early identification of NAFLD and subsequent lifestyle or medical intervention showed a 41% relative risk reduction in liver-related mortality at 5 years, compared with a strategy of not investigating these patients.^40^ Furthermore, detection of compensated cirrhosis while patients are asymptomatic and ambulatory, with enrolment into surveillance programmes, is of proven benefit to reduce mortality and morbidity,^41–43^ and also reduces the expensive healthcare utilisation of the decompensated state.

Second, early identification of chronic liver disease is likely to be cost-effective compared with traditional investigation algorithms. The investigation of liver disease in specialist centres can be expensive for the healthcare system and inconvenient to the patient. Recent economic modelling from the UK has suggested that the substitution of elastography for liver biopsy in a hospital setting would save £520 per patient investigated.^44^ Implementing these services in the community is likely to improve the health-economic benefits of early liver disease detection yet further, although future health-economic modelling studies are required to confirm this.

Lastly, there are no current medical treatments with widely proven efficacy to prevent fibrosis progression in either alcohol-related liver disease or NAFLD. Novel investigation algorithms, such as the one we have presented, identify a representative population of patients with clinically significant liver disease in whom appropriate therapies may be tested and developed. These, in conjunction with wider adoption of health-related lifestyle changes, may enable reduction of disease progression, symptomatic liver disease and liver disease-related deaths at a general population level.

## Conclusions

We have utilised a novel diagnostic approach in the general population by identifying patients with chronic liver disease risk factors and stratifying using non-invasive fibrosis biomarkers. This algorithm was feasible to implement and resulted in increased detection of asymptomatic but clinically significant liver disease compared to traditional liver enzyme-based algorithms, including a 140% increase in observed cirrhosis prevalence. Importantly, the majority of patients diagnosed with significant liver disease had normal liver function enzymes. Future studies are warranted to ensure the generalisability of our findings to other community populations, to identify the optimal biomarker algorithm for detecting clinically significant liver disease and, subsequently, the health-economic benefits of this approach compared with standard care.
